# Electrochemical degradation and saponification of porcine adipose tissue

**DOI:** 10.1038/s41598-020-76678-y

**Published:** 2020-11-27

**Authors:** Tiffany T. Pham, Anna M. Stokolosa, Pamela A. Borden, Kyle D. Hansen, Ellen M. Hong, Tatiana B. Krasieva, Ryan H. Sivoraphonh, Wesley J. Moy, Andrew E. Heidari, Lauren H. Lee, Eun-Hee Kim, Chung- Ho Sun, Wangcun Jia, Ji -Hun Mo, Sehwan Kim, Michael G. Hill, Brian J. F. Wong

**Affiliations:** 1 Beckman Laser Institute and Medical Clinic, University of California - Irvine, Irvine, CA 92612 USA; 2 Department of Chemistry, Occidental College, Los Angeles, CA 90041 USA; 3 Department of Biomedical Engineering, Beckman Laser Institute, University of California - Irvine, 1002 Health Sciences Road, Irvine, CA 92697 USA; 4 Beckman Laser Institute-Korea, Dankook University College of Medicine, Cheonan-si, Chungnam, 31116 Republic of Korea; 5 Department of Otorhinolaryngology, Dankook University College of Medicine, Cheonan-siChungnam, 31116 Republic of Korea; 6 Department of Biomedical Engineering, Dankook University College of Medicine, Cheonan-siChungnam, 31116 Republic of Korea; 7 Department of Otolaryngology - Head and Neck Surgery, School of Medicine, University of California - Irvine, Orange, CA 92868 USA

**Keywords:** Fats, Fluorescence imaging

## Abstract

Body contouring achieved via subcutaneous adipose tissue reduction has notably advanced over the past century, from suction assisted lipectomy to techniques with reduced degrees of invasiveness including laser, radiofrequency, high frequency focused ultrasound, cryolipolysis, and drug-based injection approaches. These costly techniques have focused on damaging adipocyte cell membranes, hydrolyzing triglycerides (TGs), or inducing apoptosis. Here, we present a simple, low-cost technique, termed electrochemical lipolysis (ECLL). During ECLL, saline is injected into the subcutaneous adipose tissue, followed by insertion of needle electrodes and application of an electrical potential. Electrolysis of saline creates localized pH gradients that drive adipocyte death and saponification of TGs. Using pH mapping, various optical imaging techniques, and biochemical assays, we demonstrate the ability of ECLL to induce acid and base injury, cell death, and the saponification of triglycerides in ex vivo porcine adipose tissue. We define ECLL’s potential role as a minimally-invasive, ultra-low-cost technology for reducing and contouring adipose tissue, and present ECLL as a potential new application of an emerging electrochemical redox based treatment modality.

## Introduction

The sculpting of body fat has evolved toward increasingly less invasive techniques over the past century^1^. Classic surgical excision has given way to suction lipectomy, which in turn is now being replaced by even less invasive methods such as laser lipolysis^2^, radiofrequency therapy^3^, photobiomodulation (PBM)^4^, high intensity focused ultrasound (HIFU)^5,6^, cryolipolysis^7^, and the injection of lipolytic drugs (e.g., deoxycholic acid- a bile acid)^8^. These contemporary approaches are aimed at inducing adipocyte necrosis or apoptosis, or modulating systemic lipid metabolism^9^. These minimally-invasive modalities all share in common the use of expensive technology or drugs, but offer distinct advantages of reduced surgical burden, cost, and/or recovery time. We have been exploring a novel approach based upon in situ electrochemistry to lyse adipocytes. Referred to as electrochemical lipolysis (ECLL), this method is both simple and inexpensive, requiring only a simple DC power supply and needle electrodes inserted into tumescent adipose tissue that could be manufactured for five U.S. dollars.

**Figure 1:**
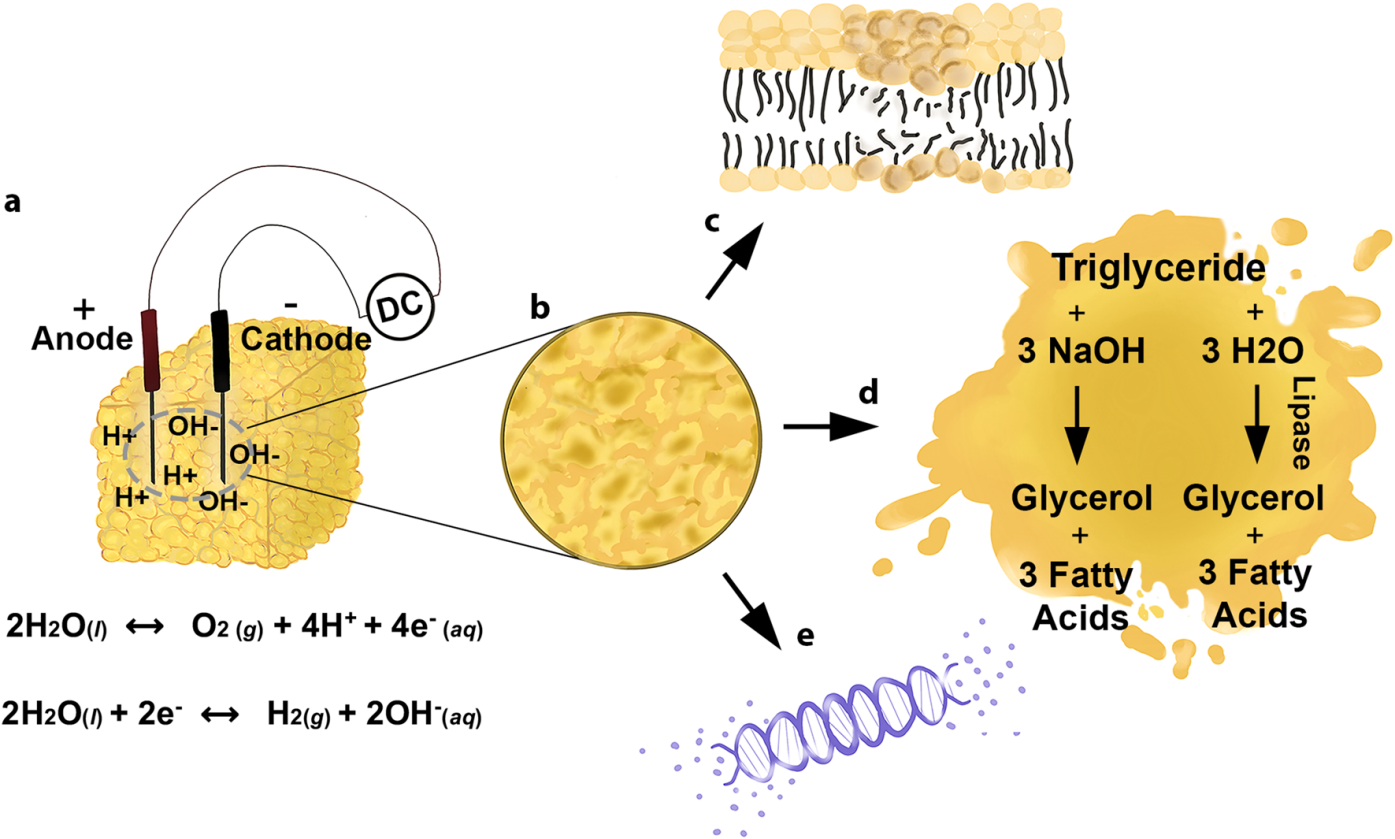
Proposed mechanism of ECLL. (**a**) Electrodes were inserted into adipose samples. Following saline injection, ECLL was performed, inducing the electrolysis of saline (water) and formation of H + and OH- ions. (**b**) ECLL destroys adipocytes via multiple mechanisms: (**c**) membrane lysis, (**d**) the saponification of triglycerides, and (**e**) nuclear degradation.

We have reported extensively on in situ electrochemical reshaping of cartilage^10–16^, and more recently discussed the potential of electrochemistry to alter collagen in skin^17^. While adipose tissue might not seem to be an obvious target for electrochemical therapy owing to its intrinsically insulating properties, injection of normal saline dramatically increases electrical conductivity and allows current to flow readily at low voltages. Under these conditions, ECLL results in controlled water electrolysis within adipose tissues, with the subsequent creation of highly localized pH gradients—hydrogen ions at the anode and hydroxide ions at the cathode (Fig. 1). As indicated by linear and non-linear imaging modalities, high-frequency ultrasonography, and biochemical assays, the electrochemical generation of acid and base triggers a series of cellular events that ultimately lead to the focal reduction of fat, through membrane lysis (Fig. 1c), saponification of triglycerides (TGs) (Fig. 1d), and nucleic acid and protein degradation (Fig. 1e). Herein, we present a detailed biophysical study of ECLL and the first report of this effect in fat by examining the cellular and molecular changes that occur in porcine adipose tissue.

## Results

### Macroscopic changes in bulk morphology

**Figure 2:**
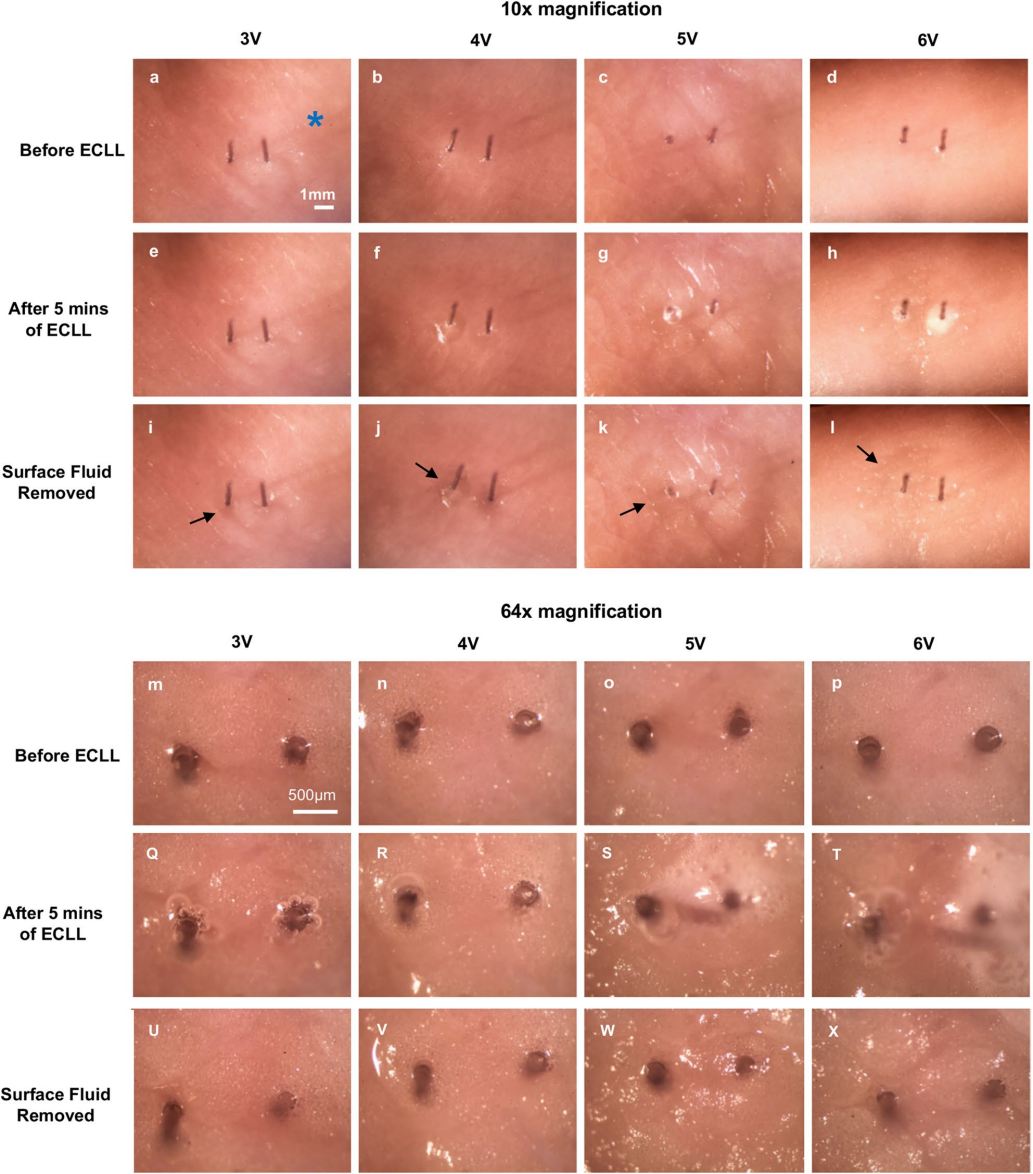
Digital photographs of adipose samples following ECLL. Adipose samples were photographed using a dissection microscope (**a**–**d**) before ECLL, (**e**–**h**) after 5 minutes (min) of ECLL, and (**i**–**l**) after the surface fluid was removed to visualize the tissue underneath the gas formation. ECLL for 5 min at increasing voltages is displayed from left to right. Within each photograph, the anode site is displayed on the left; the cathode site is displayed on the right. The black arrow demarcates boundary between affected and unaffected tissue. The blue asterisk denotes shadowing artifact from electrodes. Images were taken at 10 x (scale bar indicates 1 mm) and 64 x (scale bar indicates 500 μm) magnification.

High power digital images, acquired at the deep surface furthest away from the electrode insertion site, showed the formation of surface water, gas evolution (bubbles), and subtle surface tissue color changes accompanied by increased specular reflection surrounding the electrodes (Fig. 2). Surface changes were more apparent with increasing voltage, as seen in the increased area of change (the border of affected tissue is denoted by the black arrow in Fig. 2i-l, and with higher voltage, *i.e.*, 5 V and 6 V) (Fig. 2w, x). A similar trend was seen with increasing application time. Notably, direct palpation of samples following ECLL revealed *localized tissue softening to touch*.

### pH mapping

**Figure 3:**
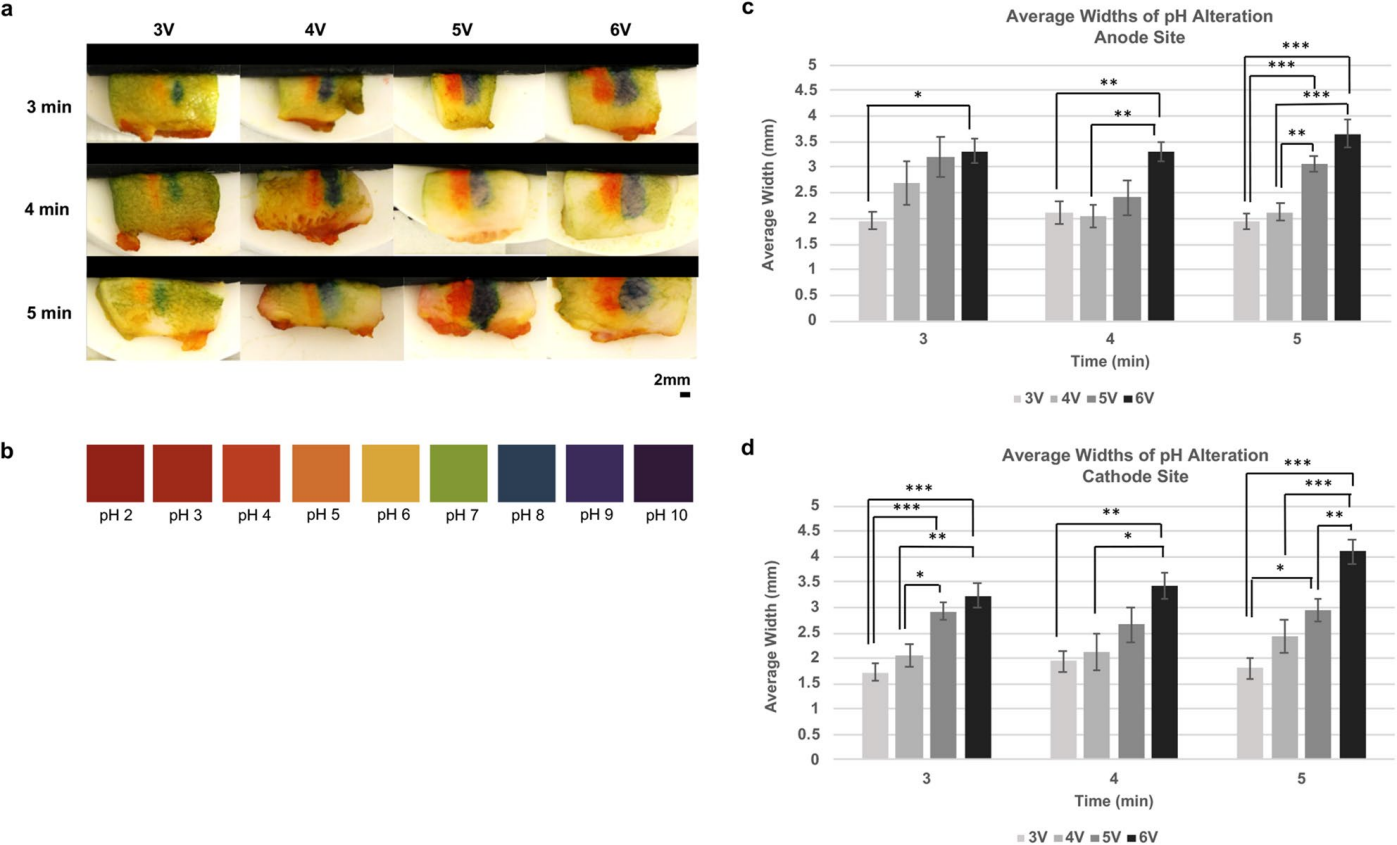
pH mapping of adipose samples following ECLL. pH dye was applied to fat samples, cross-sectioned, and imaged using digital photography. Samples are oriented with the surface of insertion site on top. Color change reflects changes in pH at anode (orange, pH 5) and cathode sites (blue, pH 8). (**a**) pH mapping is displayed at various dosimetry parameters. Scale bar indicates 2 mm. (**b**) Depicts the universal pH test chart. Bar graphs of average widths (mm) of pH alteration at the (**c**) anode site and (**d**) cathode site are plotted with increasing voltage and time. Error bars indicate standard error of the mean (SEM). Statistical significance was determined at p*≤ 0.05, p*≤ 0.01, and p***≤ 0.001.

The spatial extent of pH change on the cross sectioned adipose tissue increased with voltage and duration (Fig. 3a), with acid produced at the anode and base at the cathode. The average widths of these changes are quantified in Fig. 2c (anode) and 2d (cathode).

### Infrared thermography

Using infrared thermography, initial temperatures of tissue immediately surrounding electrodes ranged from 18.81 to 21.74 °C for all samples. At applied voltages between 3 and 6 V, the temperature increased slightly during the first minute leveling off, never rising by more than 1 °C; at 7 V, the temperature rose more than 1 °C; and at 10 V the temperature rose more than 5 °C. ECLL treatments above 6 V therefore were not evaluated in the core experiments of this study.

### High frequency ultrasonography

**Figure 4:**
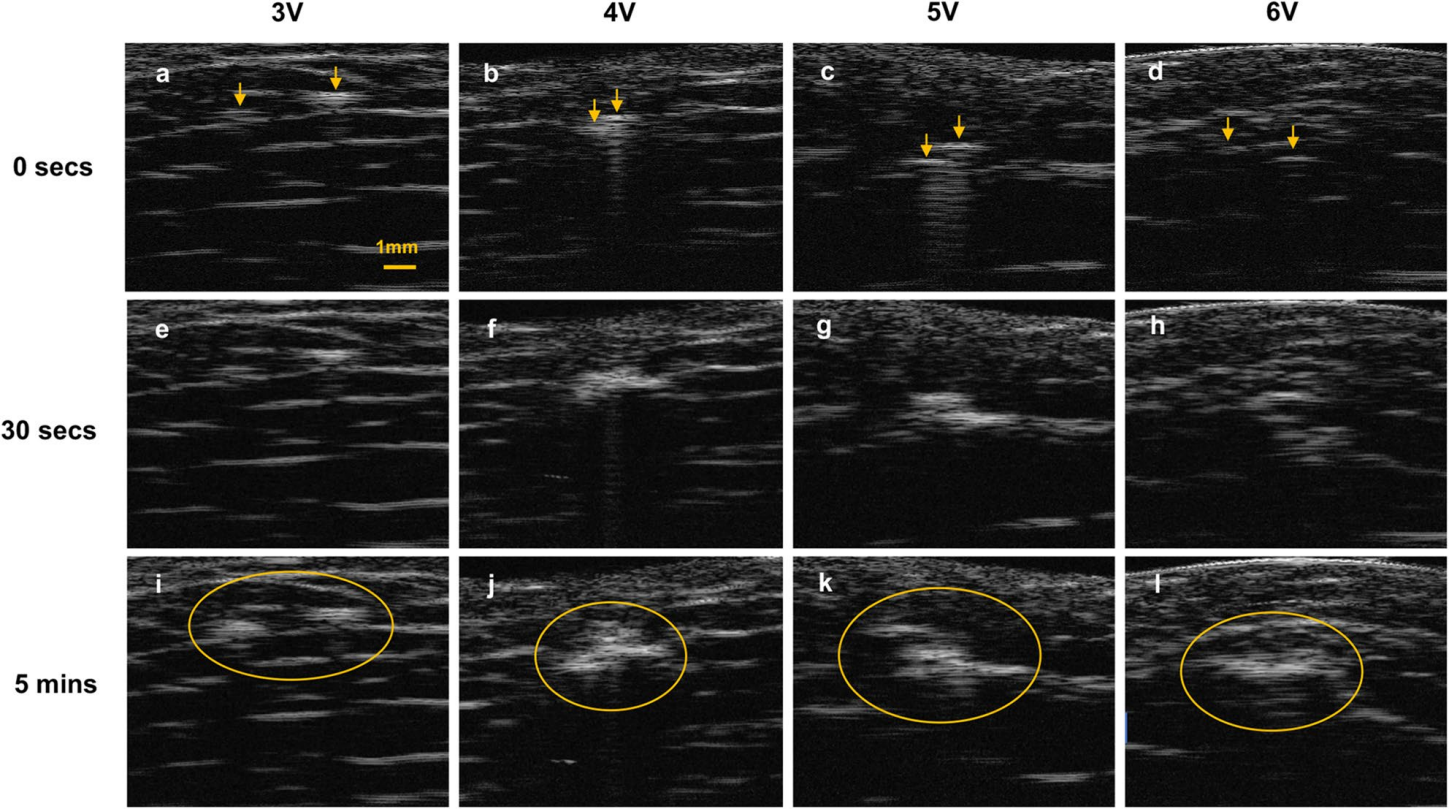
High frequency ultrasound images of adipose samples following ECLL. Adipose samples were imaged using high frequency ultrasound at (**a**–**d**) 0 s (secs) of ECLL, (**e**–**h**) 30 s of ECLL, and (**i**–**l**) 5 min of ECLL. Images of increasing voltage are displayed from left to right. Yellow arrows indicate electrodes. Yellow circles surround the hyperechoic region of gas formation.

As with photographic images, high-frequency ultrasonography during ECLL showed subtle results. Contrast may come from two sources: 1) change in intrinsic tissue mechanical properties, or 2) the evolution of molecular oxygen or hydrogen with associated microbubble formation. The latter is most likely in the case with ECLL. In Fig. 4a-d prior to ECLL, the inserted needle electrodes appeared as small hyperechoic regions with mild acoustic. With application of ECLL, there was an increase in echogenic signal around these needles, likely indicating gas formation. This was readily evident after 5 min of ECLL (Fig. 4c).

### Adipocyte morphology and lipid droplet staining

**Figure 5:**
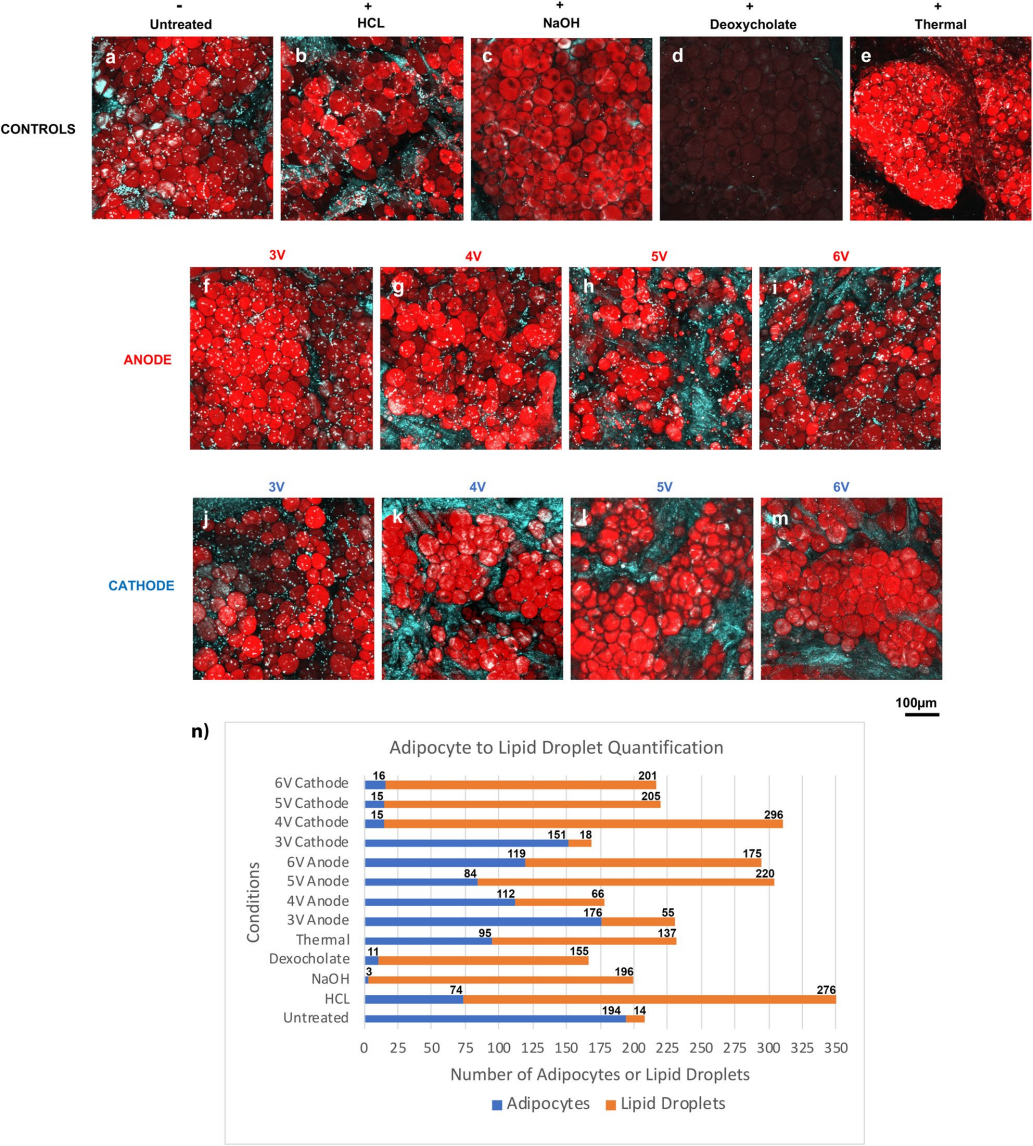
Morphology and lipid droplet assay. Representative maximum intensity projections of (**a**) untreated, (**b**) HCl, (**c**) NaOH, (**d**) deoxycholate, and (**e**) thermally treated tissues stained with Bodipy and Hoechst. ECLL was performed at increasing voltage with a constant duration of 5 min. Representative images of anode sites of (**f**) 3 V, (**g**) 4 V, (**h**) 5 V, and (**i**) 6 V and cathode sites of (**j**) 3 V, (**k**) 4 V, (**l**) 5 V, and (**m**) 6 V are depicted. Images are shown at 10 × magnification. (**n**) Adipocyte and lipid droplet quantification per condition. Number of adipocytes and lipid droplets are denoted above bars.

Bodipy 558/568 C12 lipid staining (red) demonstrated adipocyte and lipid droplet morphology at 10 × magnification in each image (Fig. 5). Hoechst 33342, which stains deoxyribonucleic acid (DNA) of all cells, was used to identify eccentric nuclei on adipocytes, represented by small blue pinpoints on red lipid staining. Collagen second harmonic generation (SHG) signal is also shown in blue, evident by long fibrils. Untreated adipocytes were of circular shape, contained eccentric nuclei, were organized into lobules separated by fibrous connective tissue septa, and constituted the bulk of the tissue volume. Variations of cell morphology and organization, Bodipy stain intensity, and presence/loss of Hoechst staining were observed in different experimental conditions [HCl, NaOH, deoxycholate, thermal (via microwave), and ECLL]. At 3 V, both the anode and cathode regions showed similar adipocyte morphology and distribution as the untreated control, suggesting a threshold parameter. For conditions 4–6 V, images of anode and cathode sites demonstrated fewer adipocytes with great heterogeneity in cell shape and the presence of lipid droplets. At the cathode, *loss of Hoechst nuclear staining* was observed. While Bodipy staining did not identify a clear relationship between 4–6 V, it is evident that cell injury and fat destruction does occur and is nonuniformly distributed. The quantification of adipocytes and lipid droplets in each condition is shown in Fig. 5n. A predominance of adipocytes in the untreated condition, a mix of adipocytes and lipid droplets in the thermally treated condition, and a predominance of lipid droplets in the HCl, NaOH, and deoxycholate was shown. The anode conditions depicted a mix of adipocytes and lipid droplets. The 3 V cathode condition showed a predominance of adipocytes, while the 4–6 V cathode conditions showed a predominance of lipid droplets.

### Live dead assay

**Figure 6:**
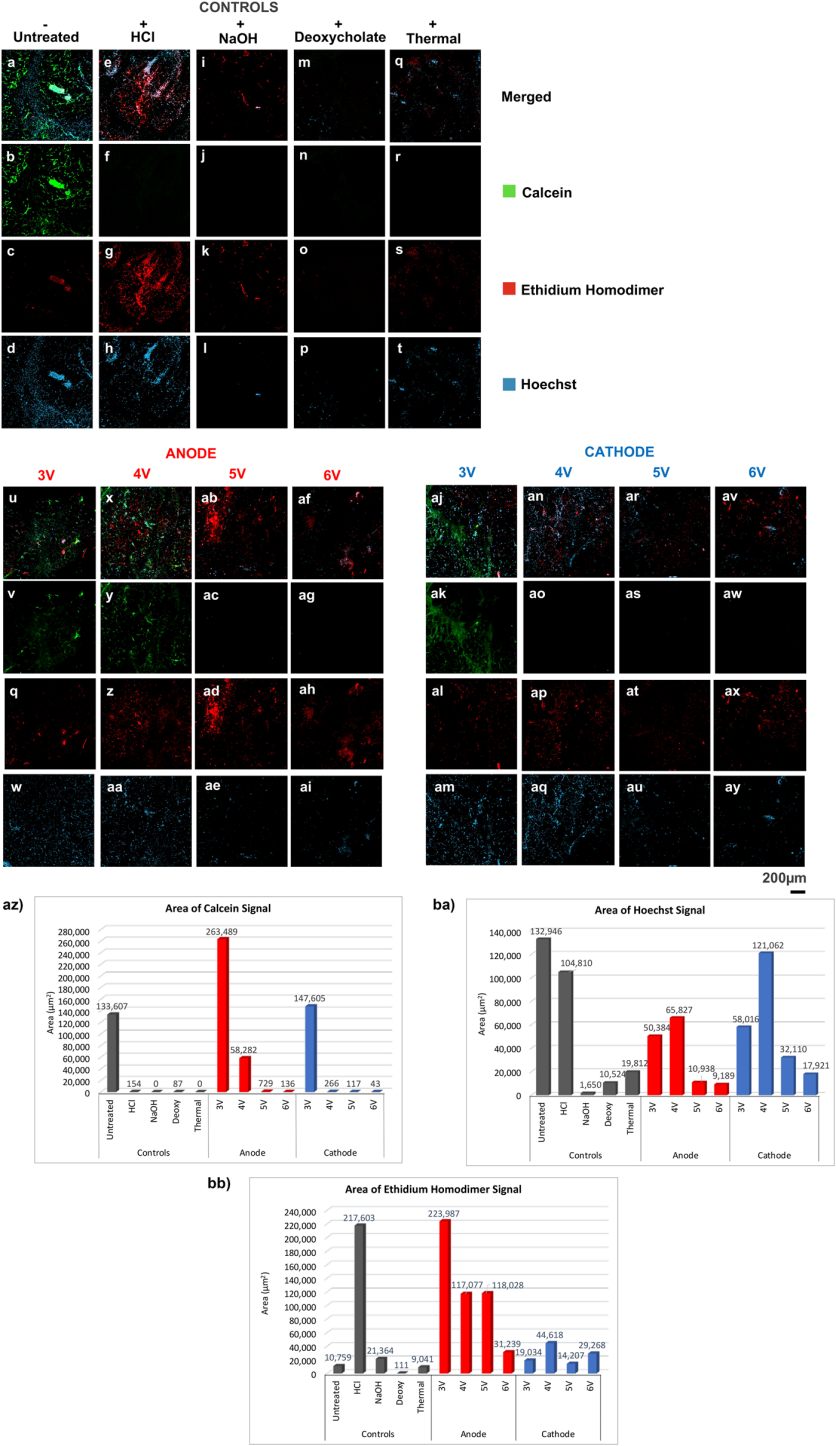
Live dead assay. Representative maximum intensity projections of adipose samples of untreated (**a**–**d**), HCl (**e**–**h**), NaOH (**i**–**l**), sodium deoxycholate (**m**–**p**), and thermal (**q**–**t**), conditions following staining with Calcein, EthD-1, and Hoechst. The images at the anode site for 5 min of ECLL at 3 V (**u**–**w**), 4 V (**x**–**aa**), 5 V (**ab**–**ae**), and 6 V (**af**–**ai**); and cathode site with identical dosimetry of 3 V (**aj**–**am**), 4 V (**an**–**aq**), 5 V (**ar**–**au**), and 6 V (**av**–**ay**) are also pictured. Images were acquired at 10 × magnification, indicating an area of 900 μm × 900 μm. Areas of (**az**) Calcein, (**ba**) Hoechst, and (**bb**) EthD-1 signal per condition.

Denoised maximum projection images of controls and ECLL treated samples in the live dead assay are shown and summarized in Fig. 6. *The presence of cytoplasmic Calcein fluorescence signal (green) and the subtraction of nuclear Hoechst signal (blue) with nuclear Ethidium Homodimer-1 (EthD-1) signal (red) indicates live cells. Alternatively, the loss of green Calcein signal and blue Hoechst signal, as well as the increased presence of red EthD-1 signal indicates cell death.* Collagen SHG signal is also shown in blue in long fibrils.

In the untreated control, there was vibrant Calcein and Hoechst signal with very little EthD-1 signal, indicating the majority of the adipocytes visualized were alive. Positive controls (HCl, NaOH, deoxycholate, thermal) showed predominantly dead adipocytes with decreased Calcein signal, and decreased nuclear Hoechst staining in the NaOH, deoxycholate, and thermal conditions. Anode sites of 3 V and 4 V conditions showed a mix of both live and dead adipocytes, while the 5 V and 6 V conditions showed a predominance of dead cells with lack of Calcein staining. Cathode sites of 3 V demonstrated a mix of live and dead cells. However, a predominance of dead cells was noted in the 4–6 V conditions with lack of Calcein staining. Interestingly, decreased Hoechst staining was noted in both 5 V and 6 V anode and cathode conditions.

Area of fluorescence signal (micron^2^) was quantified in Fig. 6az, ba, and bb. Figure 6az details decreased Calcein signal area in positive controls, 5 V and 6 V anode, and 4–6 V cathode indicating mostly dead adipocytes. In Fig. 6ba, there was less area of Hoechst signal in the NaOH, deoxycholate, thermal, and 5 V and 6 V anode and cathode conditions, indicating less nuclear staining. In Fig. bb, the HCL and 3–5 V anode conditions had the largest areas of EthD-1 staining, suggesting a large presence of dead adipocytes.

### Extracellular potassium assay for cell membrane lysis

Increases in soluble potassium levels of samples were measured via coupled plasma optical emission spectroscopy (ICP-OES) to quantify cell membrane lysis and spillage of intracellular potassium to the extracellular space. Analysis of the solutions revealed elevated potassium levels in treated samples compared to the negative control (Table 1).

**Table 1:** Extracellular potassium assay for cell membrane lysis.

Treatment method	Voltage	Applied potential^a^	[K^+^] released^b^	Volume treated (per electrode)^c^
DC voltage	3 V	–	8(3) mM	61 μL
Cathodic electrolysis^c^	–	−1 V	19(3) mM	146 μL
Anodic electrolysis^c^	–	+2 V	6(2) mM	47 μL
Negative control	–	–	< 1 mM	< 10 μL
Positive control (homogenized and sonicated tissue)	–	–	130(10) mM	1000 μL

^a^Applied potentials are reported vs. AgCl/Ag. ^b^K + determined by quantifying the 766.490 nm emission line of potassium; concentrations are reported in micromoles of potassium released per mL of tissue sample. Standard deviations are given in parentheses. ^c^Potentiostat-driven reactions were carried out for 5 min with an array consisting of three platinum electrodes (0.5 mm diameter) inserted into the tissue to a depth of 1.2 cm.

Electrochemical lipolysis performed by a potentiostat allowed evaluation of independent roles of acid- vs. base-generating reactions. Applying potentials negative of the water-reduction threshold (“cathodic ECLL”—analogous to the pH-raising cathode reaction using the DC power supply) caused significantly greater potassium release from the adipose tissue. Taking the effective intracellular [K^+^] as 130 mM—determined empirically by quantifying released potassium from tissue that had been homogenized and lysed via sonication—we calculated that cathodic ECLL (electrolysis at −1 V vs. AgCl/Ag) induced potassium release from ~ 15% of the total tissue volume. Analogous anodic ECLL (electrolysis at + 2 V) showed a smaller effect, affecting ~ 5% of the sample volume. Assuming that cell damage extends radially from the electrode surfaces to form uniform cylinders of damaged tissue, we calculated that the radius of those cylinders extended approximately 2 mm from the electrode. Notably, the resulting volumes correspond nicely to the regions of pH change following DC electrolysis (Fig. 3).

### Glycerol assay for triglyceride saponification

As TGs can be broken down into their constituent components (glycerol and free fatty acids)^18,19^, free glycerol concentrations were measured to quantify TG saponification. Standard deviations are given in parentheses. The untreated control tissue was found to have 1.4(2) mg of glycerol per liter of adipose tissue. The positive control tissue, which was homogenized and treated with 1 M methanolic KOH, was found to have 72(5) mg/L of glycerol. Cathodic ECLL performed on adjacent tissue (−1 V vs. AgCl/Ag) resulted in a free glycerol concentration of 4.3(4) mg/L, whereas anodic ECLL (+ 2 V vs. AgCl/Ag) produced a soluble glycerol concentration of 1.6(3) mg/L. Using the per-electrode volumes of treated tissue calculated in Table 1, ~ 40% of the triglyceride mass liberated during cathodic ECLL was hydrolyzed, while anodic ECLL resulted in the hydrolysis of ~ 48% of the liberated TGs (albeit a smaller total mass) into free fatty acids and glycerol.

### Histology

**Figure 7:**
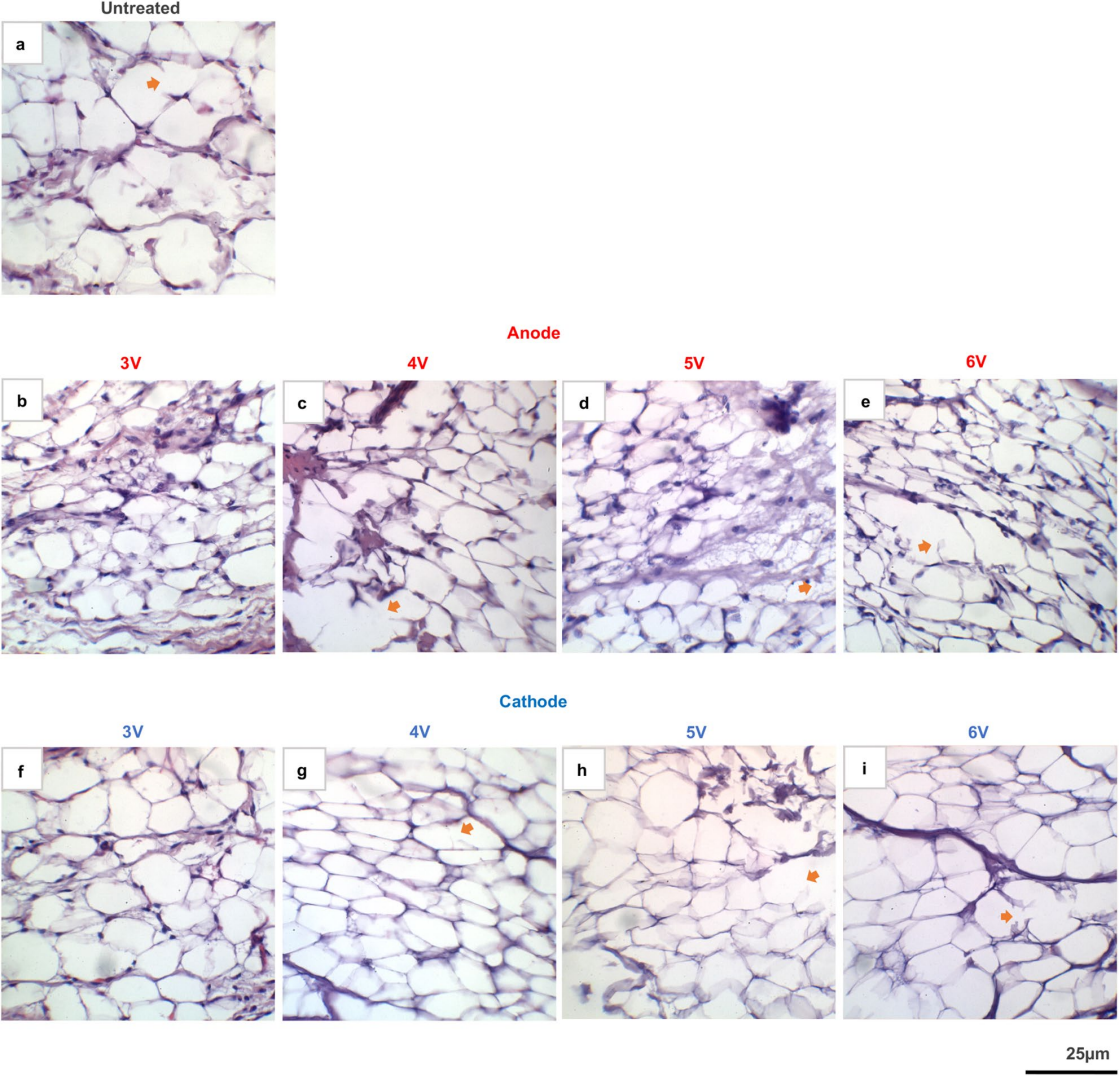
Histology of adipose samples after ECLL. H&E stained histological images of (**a**) untreated control; anode sites of (**b**) 3 V, (**c**) 4 V, (**d**) 5 V, (**e**) 6 V; and cathode sites of (**f**) 3 V, (**g**) 4 V, (**h**) 5 V, (**i**) 6 V. ECLL was performed in each of these conditions for 5 min. Orange arrows depict areas of observable cell membrane lysis possibly due to histological artifact vs. treatment effect. All images are depicted at 40 × magnification.

In untreated specimens (40x), adipocytes showed regular, spherical cell morphology with eccentric nuclei and are organized into lobules separated by connective tissue and fibroblasts (Fig. 7a). There was also some adipocyte membrane lysis noted in the untreated condition. In ECLL samples, adipocytes were inhomogeneously organized with loss of size and shape regularity, more severe at the anode site (Fig. 7b-e). Irregular membrane shape and size changes depict probable lipid depletion of the adipocyte. There was observable cell membrane lysis, mostly located adjacent to collagen fibrils. *No identifiable nuclei were observed at the cathode site* (Fig. 7f-i), indicating cell death, specifically necrosis. The effect, which is present from 4 to 6 V, was more severe with increasing voltage. The 3 V condition at both anode and cathode sites did not show obvious changes compared to the untreated control. Of note, sectioning the ECLL samples was challenging due to hardened tissue at the cathode site.

## Discussion

Current methods for fat contouring focus on bulk removal of tissue, in situ volumetric destruction (heat, cold, chemolysis etc.) with subsequent absorption of damaged cells and lipids, or the induction of apoptosis. These techniques are expensive, and vary in efficacy, spatial confinement of effect, and onset of final outcomes. For example, transcutaneous cryolipolysis may take up to six months to achieve a clinical effect, as this technique induces adipocyte apoptosis, and TGs are not hydrolyzed^20^. In the present study, we describe a novel, low-cost electrochemical alternative that simultaneously damages adipocytes and saponifies TGs. Multiple imaging modalities and biochemical assays demonstrate ECLL-induced cell membrane destruction, nuclear degradation, and TG saponification.

The ideal approach to clinical fat contouring ought to be predictable, spatially confined, simple to execute, and low cost. Through the controlled electrolysis of extracellular fluid and the injected saline, ECLL creates spatially localized damage (via acid and base damage) in adipose tissue; the volume of treated tissue can be selected by regulating the charge passed (electrical dosimetry) (Fig. 2). Because the ECLL-induced pH gradient is determined by the electric field geometry and diffusion of ions along this potential gradient and a chemical gradient, higher voltages may require shorter application times, and vice versa. Notably, the types of observed cellular injury, including lysis of the lipid rich cell membrane, nuclear degradation, and fat saponification, are expected with these pH changes^21^. For the range of potentials and time used here there is a predictable 1.5–4.5 mm lateral range of pH change surrounding each electrode. To calculate the pH changes, we used a color-segmentation algorithm that is largely unbiased. Unlike the injection of sodium deoxycholate to treat fat, ECLL may provide better control and specificity of treatment area, as it does not rely upon migration of injected agents for its effect. Furthermore, as evidenced by potentiostatic embodiments, ECLL may be feedback controlled using an operational amplifier circuit.

A major challenge working with adipose tissue is that it is extremely soft and viscoelastic, with inherent variations in tissue resistivity based on tissue hydration. Extreme effort (and practice) was required to section each sample uniformly, as the fat deforms when cut even when confined and using a sharp blade. In clinical settings, liposuction and other therapeutic treatments thus require the injection of saline to tumescence the tissue. Tumescence leads to greater rigidity, which makes the passage of a suction cannula or electrodes easier and more precise; in our techniques, it additionally serves the critical function of providing charge carriers for the electrochemical reactions. Experimentally, it is difficult to reproducibly inject 1 mL of saline precisely in the samples. Thus, there may be an inhomogeneous distribution of fluid, leading to variation in results. Modifications to sectioning and injection, such as the use of a jig, may reduce some of this variance.

Macroscopic surface changes accompanying ECLL were captured by digital photography—although the effect is more evident with the naked eye or video. Changes were subtle but included increases in tissue specular reflectance and the production of bubbles, consistent with water electrolysis. Moreover, the affected area was noticeably softened in texture. These macroscopic changes also occurred deep into the adipose specimen, localized in the areas surrounding the electrodes, and may be due to pH change or simply dehydration, although the amount of water consumed in this process was calculated to be negligible (less than 160 μg). Because the affected area increased predictably with increasing voltage and ECLL application time, dosimetry parameters can be fine-tuned readily to affect a desired volume of tissue.

Previous work showed that high frequency ultrasonography (25 MHz probe) was useful to resolve electrochemical changes in skin^17^. Our motivation for ultrasonography here was to resolve deep tissue changes penetrating the entire length of our electrodes. Ultrasonography showed likely gas evolution (presumably O_2_ and H_2_ at the anode and cathode, respectively) during ECLL, indicated by signal backscattering. The ultrasound images also demonstrated the time dependent nature of ECLL and the ability to be spatially selective based on electrode placement. However, high frequency ultrasonography did not show adipose degradation surrounding ECLL electrodes, possibly due to insufficient resolution of ultrasound to detect any subtle changes to the echogenicity of adipose tissue, which is generally hypoechoic. Furthermore, gas surrounding the electrodes may scatter sound waves and obscure any tissue changes, that which may be millimeters wide. Ultrasonography may be more applicable for clinical or in vivo uses to measure larger treatment areas or possible fat necrosis over time^22^.

Limiting voltage applications to 6 V or less ensures a minimal temperature elevation (less than 1 °C) up to at least 5 min of ECLL. Although time and voltage were directly proportional to tissue effect, at voltages above 7 V, resistive heating occurs and may cause uncontrolled injury to adjacent tissues^23,24^. Infrared thermography is well established^25^; we believe these measurements are accurate as we have extensive expertise in these methods^25–28^. Regardless, temperature at the tissue-electrode interface may be higher, though the thin layer of fat between the electrode and image plane was small (0.5–1 mm). Furthermore, these values represent average values in a region of interest (ROI) defined by the resolution of the camera. However, our thermal measurements utilized ROI selections in the appropriate anode and cathode sites, as well as the region encompassing both electrodes, and no significant differences between the sites were noted.

Bodipy staining of lipids was useful to evaluate cell morphology changes (Fig. 5). Unlike normal adipocytes (distinct, round, monotonously arranged), the specimens following 4–6 V treatments displayed irregular cell shapes, sizes, and reduced numbers. These specimens also showed the formation of subcellular lipid droplets that lack nuclear staining at the cathode site. The anode sites appeared similar to the HCl controls, while the cathode sites appeared similar to the NaOH controls. The presence of lipid droplets suggests that cell membrane integrity was compromised by ECLL and lipids were released into the interstitium. Lipid droplet formation was also observed with sodium deoxycholate treatment^29^. The use of sodium deoxycholate, an injectable drug, acts as a detergent to solubilize fat. The composition of these lipid droplets may include TGs, free fatty acids, cholesterol or cholesteryl esters^30^, and cannot be differentiated using the Bodipy staining alone. Although lipid droplets were generally identified by their smaller size and lack of nuclear staining^31^, lipid droplets of the same size as adipocytes were difficult to confidently identify. Importantly, the lack of Hoechst staining at the cathode site suggests that there was nuclear degradation with the induction of necrosis^32^; a similar effect was observed using sodium deoxycholate and NaOH. Quantitatively, 4–6 V cathode conditions showed increased number of lipid droplets, similarly to deoxycholate acid and NaOH conditions. However, statistical analysis was not able to be before due to limited data. Future studies exploring the reproducibility of this assay and findings are needed.

We have based our morphology assay on Seaman et al.’s usage of Bodipy 558/568 C12 to stain ex vivo adipose tissue to assess lipid droplet and adipocyte structure after collagenase treatment^33^. Similarly in this study, Bodipy labeled neutral lipids nonspecifically, staining both intact and non-intact adipocytes as evidenced by their live dead assay. Furthermore, Bodipy has also been used in prior literature as a synthetic precursor to study the physiological incorporation of fatty acids into phospho- and neutral lipids in live eukaryotic cells, a process that requires esterification and enzymatic activity^34–36^. Therefore, there appears to be more than one use of Bodipy to visualize lipids in various cellular or extracellular environments, as well as in in vivo or ex vivo conditions, requiring further detailed investigation of the various mechanisms of how Bodipy stains lipids. Our study findings suggest Bodipy can be used to stain lipids in both live (untreated negative control) and dead (positive control) cells, providing valuable morphological information.

The live dead assay demonstrated that ECLL lyses cell membranes at both the anode and cathode (Fig. 6). Calcein fluorescence here clearly shows loss of live adipocyte population following ECLL, more pronounced at higher voltages. The differential staining of Hoechst and EthD-1 was less obvious, possibly due to nuclear injury from the treatment conditions, causing variability in signal area and interpretation. However, further studies are required to perform statistical analysis between varying dosimetry. In addition, lack of strong nuclear staining at the cathode site indicates a necrotic pathway. Similarly Rotunda et al. performed functional staining of adipocytes and demonstrated decreased Calcein signal in adipose tissue treated with sodium deoxycholate, indicating membrane lysis^37^. In most tissue studies investigating lipolytic techniques, however, histology is the gold standard but often difficult to interpret unlike functional imaging.

Cell membrane lysis was also confirmed by measuring the increased concentration of extracellular potassium following ECLL (Table 1)^38^. As 98% of the body’s potassium is intracellular, a small release of potassium can significantly affect the concentration of measured extracellular potassium. The ratio between intracellular and extracellular potassium is approximately 40:1. A small 2.5% change in the ratio can elevate extracellular potassium concentration by 0.1 mEq/L^5^. Although there is unavoidable potassium release in the untreated controls due to mechanical shearing by sectioning and saline injection, the concentration measured from ECLL conditions was greater by more than 50%. Given the large concentration of intracellular potassium, measuring its concentration in various conditions suggests cell lysis. The majority of minimally invasive fat contouring technologies, with the exception of cryolipolysis, involve immediate cell lysis.

Free glycerol is a direct product of TG saponification—the hydrolysis of ester bonds in TGs (the storage form of fat), which occurs when reacted with a strong base. Prior literature has shown that measurements of downstream products are validated as markers of TG breakdown^39^. Following ECLL, increased free glycerol was measured with the hydrolysis of 40–48% of TG mass, indicating that saponification of TGs occurs. Although care was given to uniformly section tissue, there may be variances in sample size which may include differences in number of adipocytes and thus amount of TGs in each sample. In addition, differences in adipocyte size may contribute to potential variations in TG measurements. However, this glycerol assay remains to be an effective, functional and quantitative assessment of the ability of ECLL saponify TG, further supporting the various imaging techniques and biochemical assays used in this study to evaluate mechanisms of cell death.

H&E sections (Fig. 7) confirmed loss of nuclei at the cathode, irregular cell membrane shape and variation in shape and size, all indicating that necrosis, not apoptosis, is the cell death pathway following ECLL^32^. Unlike other mammalian cell types that undergo necrosis, adipocytes are unique in their presentation of necrosis, where often times the lack of nuclei may be the only histological feature for necrosis. Compared to the histology of adipose tissue treated with sodium deoxycholate, which showed profound blurring and dissolution of cell membranes^37^, the immediate membrane perturbations following ECLL are more subtle. However, these effects are consistent with what is known in literature of chemical injury^21,40–43^. The adipocyte lysis following ECLL occurs primarily surrounding the fibrous septa that organize lobules. However, it is important to note that membrane lysis also occurred in the untreated condition, which may be due to saline injection or sectioning artifact. The irregularity of the membranes, better visualized at the anode site than cathode site, is similar to histological images using sodium deoxycholate, radiofrequency, and ultrasound, which suggest that the irregularity demonstrates emptying of the adipocyte^44^. There are histological similarities of ECLL to adipose tissue following laser lipolysis, showing cell lysis and necrosis^45^. The therapeutic dosimetry range for ECLL-induced adipocyte necrosis may lie between 4 and 6 V. Lack of histological changes at 3 V suggest that the concentration of acid or base generated at these parameters is inadequate to produce observable changes. Limitations with histology, such as shrinkage and sectioning artifact, should be considered. In addition, adipocytes normally vary in size. Thus, inferences on a global shrinkage or swelling of tissue were difficult to perform. Challenges in sectioning the hardened cathode site can be due to a fixation artifact produced by the pH changes. Histology of tissue in base solution is not commonly performed, as most biopsies of tissue have innate chemical compound and normal physiological pH. However, the stark loss of nuclei and disrupted membranes points to the necrotic pathway of cell death induced by ECLL.

ECLL has the potential to replace scalpels, sutures, liposuction cannulas, lasers, or drugs with a molecular based approached to transiently alter the chemical structure of adipose tissue. Unlike current technologies, ECLL may be exploited as a convenient, simple, and low-cost needle-based modality to contour adipose tissue. The use of a device to provide in situ therapy may be adopted without the expense and regulatory hurdles of pharmaceutical treatments or the complexity of radiofrequency, freezing, or laser-based technologies. Unlike the injection of an acidic or basic drug, ECLL utilizes tissue as an electrochemical cell to produce byproducts that elicit similar effects to expensive pharmacologics. In addition, this highly localized method allows for spatial selectivity, dictated by the electric field and diffusion.

Our study encompasses analysis of several parameters, which clearly suggest that ECLL creates pH changes in fat that facilitate adipocyte necrosis via cell lysis, nuclear degradation, and TG saponification. There may be multiple mechanisms responsible for the observed cellular injury with ECLL. Due to the presence of chloride in interstitial fluid, transient hypochlorite species and minute quantities of chlorine gas may also be formed; these are known to be cytotoxic. Reactive oxygen species, such as hydrogen peroxide and superoxide, also likely form which in large quantities can damage DNA, cellular proteins, and lipids^46^. Further studies are needed to evaluate the presence of these chemical reactions which may be contributors to adipocyte injury.

As a novel device that takes advantage of the in situ ionic environment in biologic tissues, ECLL has the ability to generate local pH gradients to induce cell necrosis via membrane lysis, nuclear degradation, and triglyceride saponification as evident through various macroscopic and microscopic imaging modalities and biochemical assays. Electrochemical lipolysis has the potential to be a low-cost minimally invasive means for fat destruction and body contouring.

## Methods

### Overview

Electrochemical lipolysis was applied to adipose tissue and its induction of local tissue pH change, gas evolution; surface changes; as well as cell viability, lysis, and lipolytic effect was evaluated through various macro- and microscopic imaging modalities and biochemical assays.

### ECLL of adipose tissue

Adipose tissue from the cheek, submental, and neck areas was procured from freshly euthanized Yorkshire pigs used in other Institutional Animal Care and Use Committee-approved protocols. The tissue was covered in phosphate buffered saline (PBS) soaked gauze and transported in a seal proof container. Adipose tissue was sectioned into 1 by 2 cm pieces. As a medium for electrolysis and for tissue tumescence, 1 ml of normal saline (0.9% sodium chloride) was injected using a 29 AWG needle into samples. A custom acrylic jig with two platinum needle electrodes (Natus Neurology; Middleton, WI) placed 3 mm apart was inserted perpendicular into the adipose samples. ECLL using dosimetry parameters of 3–6 V exposed over 3–5 min was applied to the samples with current drawn from a power supply (Agilent, Santa Clara, CA). Current was monitored during the treatment and total charge transfer was calculated. Assuming all charge was completely utilized for splitting water, the amount of water consumed in this process was small, calculated at less than 160 μg. Without saline injection and tissue tumescence from injection, the effect of ECLL is restricted by the innate ionic environment of the adipose tissue, leading to fewer redox reactions and a decreased ECLL effect. Due to the challenging nature of obtaining punch biopsies from the small sectioned pieces, saline injection and ECLL for the microscopic procedures were carried out in unsectioned, whole harvested tissue. Saline injection also provided tissue tumescence for easy electrode insertion. To independently evaluate chemistry at each the anode and cathode sites, ECLL was performed using a potentiostat on tissue sectioned 3 cm^3^ for the potassium assay, and on 1 cm^3^ samples for the glycerol assay described below. These experiments were carried out using ICH Instruments potentiostat/galvanostat. Specimens were placed in a petri dish containing PBS/0.5 M dextrose as the electrolyte. The electrolyte volume was adjusted such that approximately 1/3 of the specimen was bathed in the solution. Potentials were held relative to an AgCl/Ag reference electrode in 3 M KCl, separated from the electrolysis solution by a Luggin capillary, and a platinum wire served as the counter electrode, and was separated from the electrolysis solution by a sintered glass frit. Of note, for each different experiment and ECLL condition below, new samples were utilized for treatment and analysis. At least three trials of each procedure were performed.

### Digital photography

Twelve adipose samples were examined and photographed using a dissection microscope (10 × and 64 × magnification) with diffuse lighting immediately before, during and immediately after ECLL (3–6 V for 5 min). Samples were imaged at the deep surface, furthest away from the side of electrode insertion. After 5 min of ECLL, surface fluid was removed via gently blotting with tissue paper and the sample was photographed once more.

### pH mapping

Following ECLL, adipose samples were sectioned with a scalpel across the centerline, determined by locus of the anode and cathode needle insertion sites. One hundred and nine individual samples were used for ECLL at 3 V to 6 V for 5 min. Two drops of halochromic pH mapping dye (Micro Essential Lab, Brooklyn, New York) were applied to the cut surface. The subsequent immediate color change was recorded using a digital single-lens reflex camera (Canon, Tokyo, Japan) with diffused even lighting. A MATLAB (MathWorks, Natick, MA) program was developed to calculate average width of pH alterations at the cut surface to estimate the extent of these perturbations as a function of electrical dosimetry. The algorithm is based upon the principles of Euclidean color distances^47,48^. Briefly, for each image, the user is first prompted to select two ROIs around the anode and cathode sites which contain the range of colors present within affected regions. These ROIs are utilized by the algorithm to correct for slight variability in color intensity and ambient lighting of the images (although great care was used to standardize the pH dying process and ambient lighting during experiments and image capture). Following the designation of the ROIs, the average LAB coordinates of each region is calculated and the Euclidean distance from the average to all pixels in the image is determined. Next, standard deviations (SDs) of the Euclidean color distances within a given ROI are calculated and all pixels with a Euclidean color distances of greater or less than one SD of the ROI are removed to generate two Boolean masks. Then hole filing and disconnected pixel removal processing steps are applied to the masks to remove pixels improperly designated as part of the ROI. Finally, the average row length in pixels is calculated for each binary mask. The measurement is converted from pixels to cm using a ruler included in the image for scale.

### Infrared thermography

An infrared thermal camera (A320, FLIR Systems, Inc.; Wilsonville, OR) was used to detect thermal changes in samples before, during, and after ECLL. Sixteen individual samples were utilized for ECLL 3–6 V for 5 min. Following electrode insertion and saline injection, excess tissue between the camera was cut away to leave only a thin layer (approximately 0.5–1 mm) of fat between electrodes and surface, in order to remove edge artifact. The camera was placed at a uniform distance of 10 cm, with the focus set between the electrode sites. Thermal image analysis was performed using MATLAB and ImageJ (National Institutes of Health, Bethesda, MD). The area of interest encompassing skin around the electrodes was selected, and time versus temperature data was generated. Evaporative cooling of samples was noted in untreated samples^49^.

### High frequency ultrasonography

Ultrasonography using a dermatologic high resolution 25 MHz clinical ultrasound device (Longport, Inc.; Arlington, VA) was used to evaluate real time depth resolved changes in adipose samples at 0 s (secs), 30 secs, and 5 min of ECLL. The adipose sample was placed in a custom jig fitted to the sample. Electrodes were inserted into the side of the sample and secured in place by the jig. The ultrasound probe was oriented orthogonal to electrodes and was fastened in place to the table using a ring stand. As coupling agents, deionized water was used between the transducer and tip of the probe, while ultrasound gel was used between the probe and adipose sample. A lateral scanning length of 15 mm over the span of 1 frame per sec was acquired at a depth of 10 mm. Twelve individual samples of ECLL 3–6 V for time points up to five mins were imaged.

### Fluorescence staining for morphology and live dead assays

Following ECLL, a new 8 mm dermatologic punch biopsy was used to excise the treated tissue. The biopsied tissues were sectioned between anode and cathode sites to expose the treated surface. For positive controls, biopsied tissues were cross sectioned, and each half (500 mm^3^) was incubated at 25 °C for 2 h in 800 μl of 1 N HCl (Thermo Fisher SA48-1, Waltham, MA), 1 N NaOH (Thermo Fisher SS266-1, Waltham, MA), or 5% sodium deoxycholate (MilliporeSigma D6750, St. Louis, MO) to emulate the effect of chemical reagents alone, or in the case of deoxycholate- the effect of an FDA approved drug. To induce thermal injury, a biopsied half was microwaved for 60 secs on high power. All specimens were washed in Hank’s Balanced Salt Solution (HBSS) three times to remove residual condition reagents then were stained with 800 μl of (10 μg/ml) Bodipy 558/568 C_12_ and (2.5 μg/ml) Hoechst 33,342 (Invitrogen Molecular Probes, Eugene, OR) at 37 °C for 45 min. Bodipy 558/568 C_12_ dye, with an affinity for nonpolar environments, was used to stain neutral lipids, including lipid droplets and adipocytes, providing a structural map of fat deposits^33,50^. Cell permeable Hoechst dye was used to stain DNA of adipocytes regardless of viability status. Another set of specimens was stained with 800 μl of (10 μg/ml) Calcein-AM, (0.75 μg/ml), Ethidium Homodimer-1 (EthD-1) (Invitrogen Molecular Probes, Eugene, OR), and (2.5 μg/ml), and Hoechst at 37 °C for 45 min. Calcein dye was used to fluorescently stain cytoplasm of live adipocytes green. Cell impermeable EthD-1 was used to stain DNA of dead cells. The use of these dyes together provides positive and negative indicators of cell viability. The subtraction of Hoechst signal and EthD-1 signal indicates live cells. Following staining, samples were washed three times with HBSS prior to imaging. Twenty-seven individual samples for various conditions were utilized total for each Bodipy/Hoechst and Calcein/EthD-1/Hoechst staining independently.

### Multiphoton and confocal fluorescence microscopy

A laser scanning microscope (LSM 510 Meta ZEISS, Jena, Germany), comprising of continuous wave narrow band lasers with center wavelength 543 nm and 488 nm for Bodipy and Calcein imaging respectively, and a two-photon laser (Chameleon Ultra, Santa Clara, CA) with pulse excitation at 800 nm for both Hoechst and collagen SHG imaging, was used to acquire z-stacks of stained control samples and samples following ECLL. A 10 × objective lens was utilized to acquire z-stacks after Bodipy/Hoechst staining (900 μm × 900 μm, 130 μm total recording depth, 5 μm step width) and Calcein/Hoechst/EthD-1 (12,728.8 μm × 12,728.8 μm, 170 μm total recording depth, 7 μm step width). Following visualization of the entire sample under the objective, multiple representative areas of adipocytes with little or no presence of collagen fibrils were selected for imaging. The cut edges of tissues and electrode insertion sites were avoided, as these areas showed mechanical injury. The excitation/emission wavelengths (nm) of the dyes are as follows: Bodipy (543/568), Hoechst (800/497), Calcein (488/515), EthD-1 (543/617). Second harmonic generation of collagen with an excitation/emission of 800/415 nm was attained.

### Quantitative analysis for morphology and live dead assays

Using maximum intensity projection images, Fiji software (ImageJ, Bethesda, Maryland) was used to count adipocytes, identified by Bodipy staining with nuclear Hoechst staining, while lipid droplets were identified by Bodipy staining with lack of nuclear Hoechst staining. Quantitative analysis of the live dead assay was performed using a custom threshold automatic fluorescence particle area analysis macro in FIJI. First, maximum intensity projection images of each individual channel (Calcein, EthD-1, and Hoechst) were subjected to a standard FIJI speckle de-noising filter and user define intensity thresholding was used to create a binary mask, wherein black areas indicate fluorescent particles. A particle is a discreet element of fluorescence that has a signal fluorescence intensity above a given threshold. A binary watershed segmentation algorithm was implemented to finely segment the individual fluorescence particles that may have otherwise been combined together during the binary intensity thresholding. Lastly, FIJI’s built in particle area analysis was performed.

### Potassium assay

Coupled plasma optical emission spectroscopy (ICP-OES) was used to measure extracellular potassium levels of untreated samples; following DC ECLL, cathodic ECLL, and anodic ECLL; and homogenization and sonication (positive control). As cytoplasmic potassium levels are substantially elevated relative to the extracellular fluid, an increase in soluble potassium following treatment was used to quantify membrane lysis. Potassium concentrations were measured at 766.490 nm with the use of PerkinElmer (Waltham, MA) Optima 7000DV ICP Optical Emission Spectrometer. The axial viewing parameters were: Plasma Flow = 15 L/min, Aux Flow = 0.2 L/min, Neb Flow = 0.60 L/min, RF Power = 1450 W, View Distance = 15 mm. Calibration solutions, 0.50, 2.0, and 10 ppm, were prepared from the dilution of PerkinElmer Pure standard (50 mg/L potassium) and 2% HNO_3_. A flush time of 10 s and sample flow rate of 1.00 mL/min were used during the measurements. Two-point background correction, peak area (7 points per peak), and survey range (766.172 – 766.808 nm) were applied. Adipose samples were rinsed at least 10 times in 5 mL of PBS/0.5 M dextrose to remove any potassium released by mechanical shearing during sample preparation. After treatment, the samples were extracted in 3 mL of PBS/0.5 M dextrose buffer for 10 min. The medium was then diluted 1:10 with 2% nitric acid for analysis. Free potassium was measured for a minimum of 5 samples for each condition.

### Glycerol assay

Free glycerol concentrations in samples were determined using a coupled enzyme assay kit (Caymen Chemicals, Ann Arbor, MI) involving glycerol kinase and glycerol phosphate oxidase, resulting in a colorimetric product proportional to the glycerol present. Ten μL Master Reaction Mix, containing assay buffer, enzyme mix, ATP and dye reagent, was added to the blank (0 glycerol standard), untreated control, positive control (homogenized and treated with 1 M methanolic KOH for TG saponification), and ECLL treated wells, containing tissue samples. At least five specimens of adjacent adipose tissue were evaluated for each run. The well plate was placed on a rotating plate, protected from light, and incubated for 20 min at 25 °C for reaction to occur. Subsequently, the absorbance at 570 nm of each well was read using a microplate reader. Concentrations were then calculated from standard curves generated from knows dilutions of pure glycerol.

### Histology

Twelve ECLL treated samples and two untreated control samples were placed in formalin and processed using standard protocol^51^. Eight-micron sections were mounted and stained with hematoxylin and eosin (H&E). Light microscope images at 40 × magnification were acquired of representative sections of ECL sites and controls.

### Statistical analysis

One-way Analysis of Variances (ANOVA) with post hoc Tukey tests were performed to statically compare pH alteration maps at each dosimetry parameter. Two tailed paired t-tests were used to compare pH alteration differences between anode and cathode sites. Statistical significance was determined at a value of p*≤ 0.05, p*≤ 0.01, and p***≤ 0.001. All data is expressed as mean ± SEM. IBM SPSS Statistics for MacOs, Version 24 (IBM Corp., Armonk, NY) was used for statistical analysis.

### Ethical approval and informed consent

Our study does not include live vertebrates, humans or humans samples.

## Supplementary information

Supplementary information.

## Data Availability

Requests for supporting data may be addressed to the corresponding author.

## Code availability

The pH mapping and particle area fluorescence analysis codes are available in Supplementary Information. The code for ECLL current monitoring can be provided upon request.
