# Supplementary material for: Electrochemical degradation and saponification of porcine adipose tissue
Source: Sci Rep. 2020 Nov 27;10:20745. doi: 10.1038/s41598-020-76678-y (PMC7695738; doi:10.1038/s41598-020-76678-y)
Supplement: Supplementary file 1 — Supplementary information. [file 41598_2020_76678_MOESM1_ESM.pdf]

Supplementary Information

Manuscript Title: Electrochemical Degradation and Saponification of Porcine Adipose Tissue

Authors: Tiffany T. Pham, MD MS; Anna M. Stokolosa, BA; Pamela A. Borden, BS; Kyle D. Hansen, BS; Ellen M. Hong, BA; Tatiana B. Krasieva, PhD; Ryan H. Sivoraphonh, BS; Wesley J. Moy, PhD; Andrew E. Heidari, PhD; Lauren H. Lee, BS; Eun-Hee Kim, MS; Chung-Ho Sun, PhD; Wangcun Jia, PhD; Ji-Hun Mo, MD PhD; Sehwan Kim, PhD; Michael G. Hill, PhD; Brian J. F. Wong, MD PhD

MATLAB pH Mapping (Colorimetric Segmentation) Code

```
%% Manual pH Mapping Script

%% Directions for Impoly
%   https://www.mathworks.com/help/images/ref/impoly.html

%% Init-
clc;
clear;
close all;


%% Important Variables
rect_positions = zeros([1,4,3]); % Positions of Rectanges

%% Selecting File
startingFolder = cd;

defaultFileName = fullfile(startingFolder, '*.*');
[baseFileName, folder] = uigetfile(defaultFileName, 'Select an image');
if baseFileName == 0
	% User clicked the Cancel button.
	return;
end
pic_name = fullfile(folder, baseFileName);
myImage = imread(pic_name);
title('Crop Image');
figure(1);
imshow(myImage)
[myImage,rect] = imcrop(myImage);
rect_positions(:,:,1) = rect;
```

```
figure(1);
imshow(myImage);

%% Segmenting / Create Masks
blue_mask = impoly();
wait(blue_mask);

red_mask = impoly();
wait(red_mask);

blue_mask = blue_mask.createMask();
red_mask = red_mask.createMask();

%% Segmentation of Masks
red = zeros(size(myImage));
blue = zeros(size(myImage));

blue_mat = repmat(blue_mask, [1,1,3]);
red_mat = repmat(red_mask, [1,1,3]);
blue = myImage .* uint8(blue_mat);
red = myImage .* uint8(red_mat);


figure();
imshow(blue);
figure();
imshow(red);

%% Calculate Statistics

% Converts fron logical to uint8
red_mask = uint8(red_mask);
blue_mask = uint8(blue_mask);


% Sums values along columns
red_mean = sum(red_mask,2);
blue_mean = sum(blue_mask,2);

% Converts rows with 0 to NaN
red_mean(red_mean == 0) = NaN;
blue_mean(blue_mean == 0) = NaN;

% Calculates Mean of row sums
```

```
red_mean = nanmean(red_mean);
blue_mean = nanmean(blue_mean);

% Calculates Total number of pixels in data
red_area = sum(sum(red_mask));
blue_area = sum(sum(blue_mask));

% Stores Data as a Cell
data = cell(1,5);
data{1,1} = baseFileName;
data{1,2} = red_mean;
data{1,3} = blue_mean;
data{1,4} = red_area;
data{1,5} = blue_area;



%% Export Statistics
% Notes:
%     If Invalid File ID error: Close Spreadsheet.

[baseFileName1, folder1] = uigetfile({'*.csv';'*.*'}, 'Select an Excel File' );
if baseFileName1 == 0
        % User clicked the Cancel button.
        return;
end
csv_name = fullfile(folder1, baseFileName1);
fid = fopen(csv_name,'a');
fprintf(fid,'%s, %f, %f, %f, %f\n',data{1,:});
fclose(fid);
```

Fiji (ImageJ) Particle Area Analysis Macro for Live Dead Assay

```
run("Bio-Formats Importer")
run("Split Channels");

for (i = 1; i < 4 ; i++) {

	waitForUser("Please Select an Image for processing");
	imageID = getImageID();
	selectImage(imageID);
	Dialog.create("Please rename your image");
	Dialog.addString('File Name', '');
	Dialog.show();
	rename_var = Dialog.getString();
	rename(rename_var);
	run("Z Project...", "projection=[Max Intensity]");
	run("Despeckle");
	run("Brightness/Contrast...");
	waitForUser("Please Set Your Brightness and Contrast");
	run("Duplicate...");
	saveAs('jpeg');
	run("Threshold...");
	waitForUser("Please Set Your Threshold");
	setOption("BlackBackground", false);
	run("Convert to Mask");
	run("Invert");
	run("Watershed");
	saveAs('jpeg');
	run("Analyze Particles...", "size=0-Infinity include add in_situ");
	roiManager("Select all");
	roiManager("Measure");
}
run("Image Calculator...")
run("Analyze Particles...", "size=0-Infinity include add in_situ");
roiManager("Select all");
roiManager("Measure");
saveAs('jpeg');

run("Merge Channels...");
```
